# SARS-CoV-2 infection induces hyaluronan production *in vitro* and hyaluronan levels in COVID-19 patients relate to morbidity and long-term lung impairment: a prospective cohort study

**DOI:** 10.1128/mbio.01303-24

**Published:** 2024-09-20

**Authors:** Urban Hellman, Ebba Rosendal, Joakim Lehrstrand, Johan Henriksson, Tove Björsell, Alfred Wennemo, Max Hahn, Björn Österberg, Luiza Dorofte, Emma Nilsson, Mattias N. E. Forsell, Anna Smed-Sörensen, Anna Lange, Mats G. Karlsson, Clas Ahlm, Anders Blomberg, Sara Cajander, Ulf Ahlgren, Alicia Lind, Johan Normark, Anna K. Överby, Annasara Lenman

**Affiliations:** 1 Department of Clinical Microbiology, Umeå University, Umeå, Sweden; 2 Department of Public Health and Clinical Medicine, Umeå University, Umeå, Sweden; 3 The Laboratory for Molecular Infection Medicine Sweden (MIMS), Umeå University, Umeå, Sweden; 4 Umeå Centre for Molecular Medicine (UCMM), Umeå University, Umeå, Sweden; 5 Department of Molecular Biology, Umeå Centre for Microbial Research (UCMR), Umeå University, Umeå, Sweden; 6 IceLab, Umeå University, Umeå, Sweden; 7 Centre for Clinical Research and Education, Region Värmland, Karlstad, Sweden; 8 Division of Immunology and Allergy, Department of Medicine Solna, Karolinska Institutet, Karolinska University Hospital, Stockholm, Sweden; 9 Department of Laboratory Medicine, Faculty of Medicine and Health, Örebro University, Örebro, Sweden; 10 Department of Infectious Diseases, Faculty of Medicine and Health, Örebro University, Örebro, Sweden; 11 Department of Surgical and Perioperative Sciences, Umeå University, Umeå, Sweden; 12 Wallenberg Centre for Molecular Medicine, Umeå University, Umeå, Sweden; Johns Hopkins University, Baltimore, Maryland, USA; University of Virginia, Charlottesville, Virginia, USA

**Keywords:** COVID-19, hyaluronan, hyaluronic acid, SARS-CoV-2, lung impairment, 3D-lung model

## Abstract

We previously demonstrated that the lungs of deceased COVID-19 patients were filled with a clear hydrogel consisting of hyaluronan (HA). In this translational study, we investigated the role of HA at all stages of COVID-19 disease to map the consequences of elevated HA on morbidity and identify the mechanism of severe acute respiratory syndrome coronavirus 2 (SARS-CoV-2)-induced HA production. A reduced alveolar surface area was observed in the lungs of deceased COVID-19 patients compared to healthy controls, as visualized by a 3D rendering of lung morphology using light-sheet fluorescence microscopy. We confirmed the presence of HA in lung biopsies and found large quantities of proinflammatory fragmented HA. The association of systemic HA in blood plasma and disease severity was assessed in patients with mild (WHO Clinical Progression Scale, WHO-CPS, 1–5) and severe COVID-19 (WHO-CPS, 6–9) during the acute and convalescent phases and related to lung function. We found that systemic levels of HA were high during acute COVID-19 disease, remained elevated during convalescence, and were associated with a reduced diffusion capacity. *In vitro* 3D-lung models, differentiated from primary human bronchial epithelial cells, were used to study the effects of SARS-CoV-2 infection on HA metabolism, and transcriptomic analyses revealed a dysregulation of HA synthases and hyaluronidases, both contributing to increased HA in apical secretions. Furthermore, corticosteroid treatment reduced the inflammation and downregulated HA synthases. Our findings demonstrate that HA plays a role in COVID-19 morbidity and that sustained elevated HA concentrations may contribute to long-term respiratory impairment.

**IMPORTANCE:**

This study provides insights into the role of hyaluronan (HA) in the severity and long-term impact of COVID-19 on lung function. Through extensive morphological examination of lung tissues and a multicenter study, we identified that HA levels are significantly elevated in COVID-19 patients, correlating with a reduced lung diffusion capacity during convalescence. Using a 3D-lung model, we further uncovered how severe acute respiratory syndrome coronavirus 2 (SARS-CoV-2 infection causes a dysregulated HA metabolism, leading to increased HA production. Our findings provide valuable insights into the pathogenesis of SARS-CoV-2 and suggest that targeting HA metabolism could offer new therapeutic avenues for managing COVID-19, particularly to prevent long-term lung impairment. Additionally, HA holds potential as a biomarker for predicting disease severity, which could guide personalized treatment strategies.

## INTRODUCTION

The COVID-19 pandemic, caused by the severe acute respiratory syndrome coronavirus 2 (SARS-CoV-2), has, as of early 2024, caused more than 7 million reported deaths globally (according to WHO), a figure that likely underestimates the actual number of fatalities attributable to the disease (1). The clinical manifestations of COVID-19 range from asymptomatic or mild disease with symptoms from the upper respiratory tract to severe pneumonitis with acute respiratory distress syndrome (ARDS) and multiorgan failure. Several pathophysiological mechanisms have been described as contributing to respiratory failure in COVID-19, including hyperinflammation with disturbed coagulation, leading to disseminated pulmonary microthrombi as well as diffuse alveolar damage, alveolar septal fibrous proliferation, and pulmonary consolidation (2, 3).

Hyaluronan (HA) is a glycosaminoglycan that constitutes an important structural component of the extracellular matrix in tissues. Through interactions with cell-surface receptors, HA also regulates cellular functions, such as cell-matrix signaling, cell proliferation, angiogenesis, and cell migration (4). HA is often present in its high molecular weight structure in healthy tissue and has an anti-inflammatory effect (5). In contrast, low molecular weight HA has been shown to have a pro-inflammatory effect when HA fragments bind to toll-like receptors and induce NF-κB signaling (6). HA also influences the development of fibrosis by affecting fibroblast proliferation, differentiation, and motility (7). In addition, HA has a very high water-binding capacity, with the ability to occupy large, hydrated volumes up to 1,000 times its molecular mass, which can promote edema formation (4). Accumulation of HA is associated with ARDS (8), and we and others have recently shown increased levels of HA in the lungs of deceased COVID-19 patients (9, 10) as well as elevated plasma levels of HA in severe cases (11–14). Anti-inflammatory treatment with the corticosteroid dexamethasone results in lower mortality among hospitalized COVID-19 patients (15). Corticosteroids are known to be effective in reducing HA levels in other inflammatory syndromes (16, 17); thus, clearance of HA may be an important consequence of corticosteroid treatment in the resolution of COVID-19 (9). We recently found that severe COVID-19 is an important risk factor for impaired respiratory function, characterized by a decrease in diffusion capacity (DL_CO_), 3–6 months after the infection (18). However, if the lung function correlates with systemic HA is currently not clear.

Here, we set out to investigate HA at all stages of COVID-19 disease, from acute infection to convalescence in mild, severely ill, and fatal cases. Our aim was to understand molecular mechanisms involved in the pathological overproduction of HA in disease and pathogenicity. In addition, we established an *in vitro* 3D-lung model to investigate the impact of SARS-CoV-2 infection on HA metabolism as well as the role of corticosteroid treatment in defining molecular disease mechanisms.

## RESULTS

### Morphological differences induced by COVID-19 visualized by light sheet fluorescent microscopy

**Fig 1:**
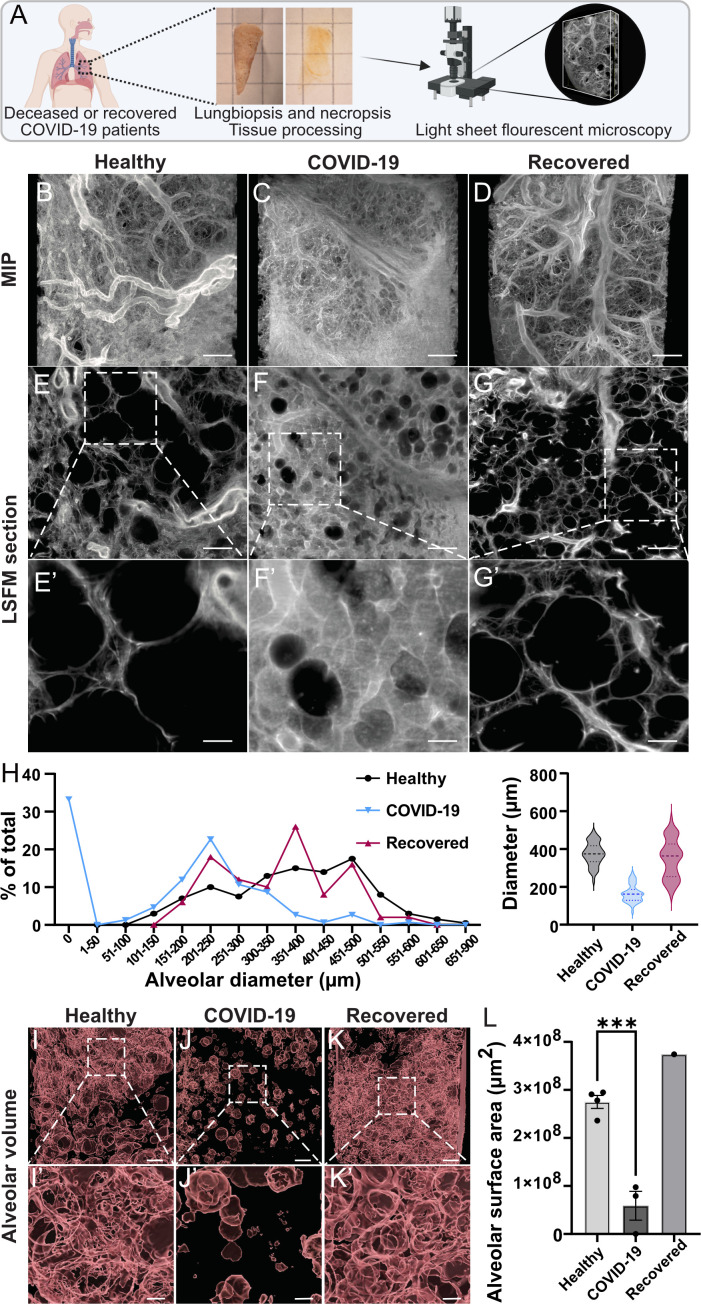
Light sheet fluorescent microscopy of biopsies from deceased COVID-19 patients displays a reduced alveolar surface. (**A**) Schematic overview of LFSM procedure with tissue biopsies from healthy donors (*n* = 4), deceased COVID-19 patients (*n* = 3), and recovered COVID-19 patient (*n* = 1). (**B–D**) Maximum intensity projections of one representative biopsy from each patient group. Scale bar is 500 µm. (**E–G**) LSFM sections and magnifications showing the alveolar structure in each patient group. Scale bar in panels E–G is 500 µm and 100 µm in panels E′–G′. (**H**) Size distribution of alveolar diameter measured in LSFM sections and shown as the percentage of total alveoli within each patient group. Ten random diameter measurements in five different z planes were done on each patient, in total 50 measurements/patient (healthy donors *n* = 4, COVID-19 *n* = 3, and recovered *n* = 1). (**I–K**) Iso-surfaced empty space in the lung biopsies as a proxy for alveolar volume. Scale bar in panels I–K is 400 µm and 100 µm in panels I′–K′. (**L**) Quantification of the surface area surrounding the empty space as a proxy for alveolar surface area. Mean values and SEM are shown; statistical significance was calculated by unpaired *t*-test (***P < 0.001).

We have previously shown that the lungs of fatal COVID-19 patients are filled with HA (9). However, it is not clear what consequences HA has on the structural integrity of the lung and the alveolar volume. To address this, we set out to morphologically determine the three-dimensional organization within lung biopsies with light sheet fluorescence microscopy (LSFM). These biopsies originated from three deceased COVID-19 patients and four healthy donors who underwent lung resection. In addition, we included one severe COVID-19 patient who underwent lung resection 10 weeks post-intensive care treatment as an individual case, here referred to as recovered. The lung biopsies were processed, cleared, and subjected to LSFM visualizing the autofluorescence (Fig. 1A). Major differences in the morphology of the lung were detected by maximum intensity projection (Fig. 1B through D; Fig. S1; Movies S1 to S3). The lungs of fatal COVID-19 patients were considerably denser compared to the healthy controls and recovered lungs. Optical sections of the tissue revealed thin alveolar walls in the controls and recovered, whereas the distances between the alveoli in COVID-19 lungs were larger (Fig. 1E through G). Size distribution measurements of the alveoli showed generally smaller diameters in the COVID-19 lungs compared to control and recovered lungs (Fig. 1H). The number of empty alveoli (seen as dark areas in Fig. 1E through G) was also fewer in the COVID-19 lungs as visualized in the surface rendering of the alveolar space (Fig. 1I through K). The alveolar surface was quantified to better understand the capacity of the gas exchange in the samples. The isosurfaces of the alveoli from Fig. 1I through K were determined and showed a major loss of alveolar surface in the COVID-19 lungs compared to the healthy controls (Fig. 1L). Worth noting is that the lung morphology seen in the recovered COVID-19 patient showed high similarity to the healthy controls.

**Fig 2:**
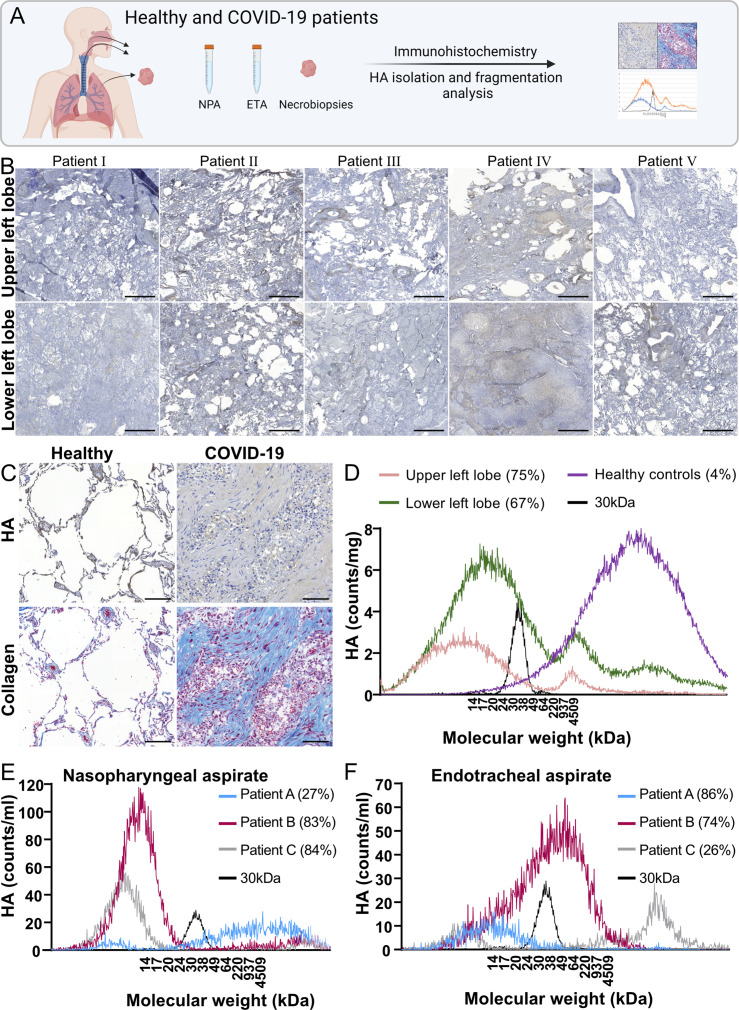
Lung necropsies and lung aspirates from severe COVID-19 patients show large amounts of hyaluronan and a high degree of HA fragmentation. (**A**) Schematic overview of the collection of patient samples for HA analysis. NPA, nasopharyngeal aspirate; EPA, endotracheal aspirate. (**B**) Lung biopsies from the upper and lower left lobes of five deceased COVID-19 patients were stained for HA using a HA binding protein (brown) and nuclei staining (blue). Scale bar is 1,000 µm. (**C**) Collagen and HA staining in lung biopsies from healthy lung tissue (scale bar is 200 µm) and from a deceased COVID-19 patient (scale bar is 100 µm). The COVID-19 lung biopsy shows areas with no alveoli structures. Upper row shows HA staining in brown and nuclei staining in blue, lower row shows collagen staining in blue, cytoplasm in pink, and nuclei in dark brown. (**D**) HA was isolated from the lung biopsies in panel B (*n* = 5) and a size distribution analysis was performed with gas-phase electrophoretic mobility molecular analysis (GEMMA). Shown is an average of all five patients. Low molecular weight HA with a known molecular size of 30 kDa was included as a control. The percentage of fragmented HA (<100 kDa) in each lobe is shown in brackets. HA was also isolated and subjected to size analysis by GEMMA from (E) nasopharyngeal aspirate and (F) endotracheal aspirate collected from three severe COVID-19 patients and displayed individually for each patient. The percentage of fragmented HA (<100 kDa) for each patient is shown in brackets.

To confirm the presence of HA in alveoli and to see if there is a difference in HA accumulation between the upper and lower lung lobes, histochemistry was performed on five COVID-19 necropsies (Fig. 2A and B). We did not observe a major difference in HA accumulation between patients or lung regions; however, extensive HA accumulations were seen in COVID-19 necropsies compared to healthy controls (Fig. 2B; Fig. S2A). Within the lung biopsies, we found distinct areas with various stages of alveolar disruptions, including (i) intact alveoli with thickened HA-containing walls, (ii) alveoli filled with secretions of HA, and finally (iii) completely obliterated alveoli replaced by collagen coils, serving as a marker for irreversible lung damage (Fig. 2C; Fig. S2B).

### Severe COVID-19 induces fragmentation of HA in lung tissue and aspirates

As fragmented low molecular weight HA increases at sites of active inflammation and displays a proinflammatory activity, HA fragmentation likely contributes to the disease progression of COVID-19. To investigate this, HA was extracted from five necropsies derived from the upper and lower left lobes of five COVID-19 patients and compared with samples from five healthy donors. The isolated HA was subjected to size determination analysis by gas-phase electrophoretic mobility molecular analysis (GEMMA). The healthy controls mainly contained high molecular weight HA (Fig. 2D), which is known to have anti-inflammatory properties. In contrast, both the upper and lower lung lobes from COVID-19 patients displayed highly fragmented HA (Fig. 2D; Fig. S2C through G). Although we did not observe any major differences in the abundance of HA with histochemistry between the upper and lower lung lobes (Fig. 2B), we found higher amounts of both total HA and fragmented HA in the lower parts of the lung with GEMMA (Fig. 2D; Fig. S2H). Interestingly, despite the higher abundance of HA in the lower lung lobes, no notable difference was seen in the degree of fragmentation between the upper and lower lobes (Fig. S2I). Since fragmented HA induces a proinflammatory state and initiates the migration of immune cells (19), we analyzed the presence of neutrophils in the lungs by staining for elastase. We detected major neutrophil infiltration in COVID-19 lungs compared to uninfected controls (Fig. S3). Next, nasopharyngeal aspirates (NPAs) and endotracheal aspirates (ETAs) collected from three additional COVID-19 patients were analyzed to investigate the fragmentation process of HA during ongoing severe disease. All patients displayed fragmented HA; however, in contrast to the fragmentation in the necropsy samples, there were large individual variations between the total amount of HA as measured by the area under the curve (NPA: 444, 1,107, and 500; ETA: 161, 977, and 272) and degree of fragmentation (NPA: 27%, 83%, and 84%; ETA: 86%, 74%, and 26%) (Fig. 2E and F).

### Hyaluronan levels in plasma increase in severe COVID-19 and are associated with a long-term reduction in lung function

The finding that HA accumulated in the lungs and aspirates of severely diseased patients prompted us to measure the systemic levels of HA. We performed this in a cohort of mild and severely ill patients during acute disease and convalescence. Our objective was to investigate if plasma concentrations of HA could be used as a biomarker for disease severity.

Patients with COVID-19 infection classified as severe, based on the requirement of high-flow nasal oxygen treatment and/or admission to the intensive care unit (ICU) corresponding to the WHO Clinical Progression Scale (WHO-CPS) 6–10 (20), were matched according to age to patients with mild COVID-19 (WHO-CPS 1–5). In total, 103 individuals were selected; 37 patients were classified as severe and 66 patients as mild. The demography and clinical characteristics are presented in Table 1. The groups differed significantly in BMI, obesity, minimum levels of hemoglobin, maximum levels of C-reactive protein, white blood cell count, and neutrophil count. No significant differences were observed when it came to comorbidities at baseline. Thirty-two (86.5%) patients in the severe group and four (6.1%) in the mild group received corticosteroid treatment (*P* < 0.001). Most of these patients, 28 in the severe and 2 in the mild group, received the first dose before the first sampling time point.

**TABLE 1:** Demography and characteristics of COVID-19 patients^*a*^^,^^*b*^

Patient characteristics	Severe (*N* = 37)	Mild (*N* = 66)	*P*-value
Demography			
Age (years)	58.0 (46.0–64.0)	54.5 (43.3–61.5)	0.584
Sex (female) *N* (%)	11 (29.7)	31 (47.0)	0.112
BMI	30.5 (27.6–33.3)	26.3 (24.0–28.9)	<0.001
Obesity, *N* (%)	20 (54.1)	15 (22.7)	0.002
Comorbidities			
Diabetes, *N* (%)	4 (10.8)	5 (7.6)	0.719
Hypertension, *N* (%)	12 (32.4)	17 (25.8)	0.500
Cardiovascular disease,^*c*^ *N* (%)	4 (10.8)	11 (16.7)	0.564
Chronic pulmonary disorder,^*d*^ *N* (%)	8 (21.6)	11 (16.7)	0.600
Autoimmune disease, *N* (%)	2 (5.4)	5 (7.6)	1.000
Charlson comorbidity index	0 (0–1.0)	0 (0–0.8)	0.584
Smoking^*e*^			0.384
Smoker, *N* (%)	1 (2.8)	1 (1.7)	
Ex-smoker, *N* (%)	13 (36.1)	14 (23.7)	
Laboratory findings			
C-reactive protein, max (mg/L)^*f*^	166 (84.5–278)	12 (3.1–76.0)	<0.001
Hemoglobin, min (g/L)^*g*^	126 (105–133)	134 (121–144)	0.004
White bloodcell count, max (10e9/L)^*g*^	11.9 (8.9–16.1)	6.9 (5.4–8.0)	<0.001
Neutrophil count, max (10e9/L)^*g*^	9.3 (6.0–12.3)	4.15 (2.9–5.1)	<0.001
Lymphocyte count, max (10e9/L)^*g*^	2.3 (1.7–2.3)	2.05 (1.7–2.4)	0.061
Platelet count, min (10e9/L)^*h*^	193 (167–233)	187 (134–232)	0.251
P-creatinine, max (μmol/L)^*g*^	79 (68.0–98.0)	77.5 (70.3–90.0)	0.975
Interleukin-6 max (ng/L)^*i*^	141 (27.3–353)	22 (14.3–35.8)	0.002
Treatment and respiratory support			
Corticosteroid treatment, *N* (%)	32 (86.5)	4 (6.1)	<0.001
Remdesivir treatment, *N* (%)	15 (40.5)	3 (4.5)	<0.001
Invasive mechanical ventilation, *N* (%)	8 (21.6)	0 (0)	<0.001

^*a*^ Descriptive data of the patient cohort. All values are presented as median and interquartile range if not otherwise stated.

^*b*^ Differences between groups analyzed by Mann-Whitney *U*-test (continuous variables) and Fisher’s exact test (dichotomous variables), except when otherwise stated. No correction for multiple testing was performed.

^*c*^ Including heart failure, ischemic heart disease, peripheral arterial insufficiency, deep venous thrombosis, and pulmonary embolism.

^*d*^ Including asthma and chronic obstructive pulmonary disease.

^*e*^ *N* = 95, analyzed by chi-square test.

^*f*^ *N* = 91.

^*g*^ *N* = 93.

^*h*^ *N* = 92.

^*i*^ *N* = 66.

**Fig 3:**
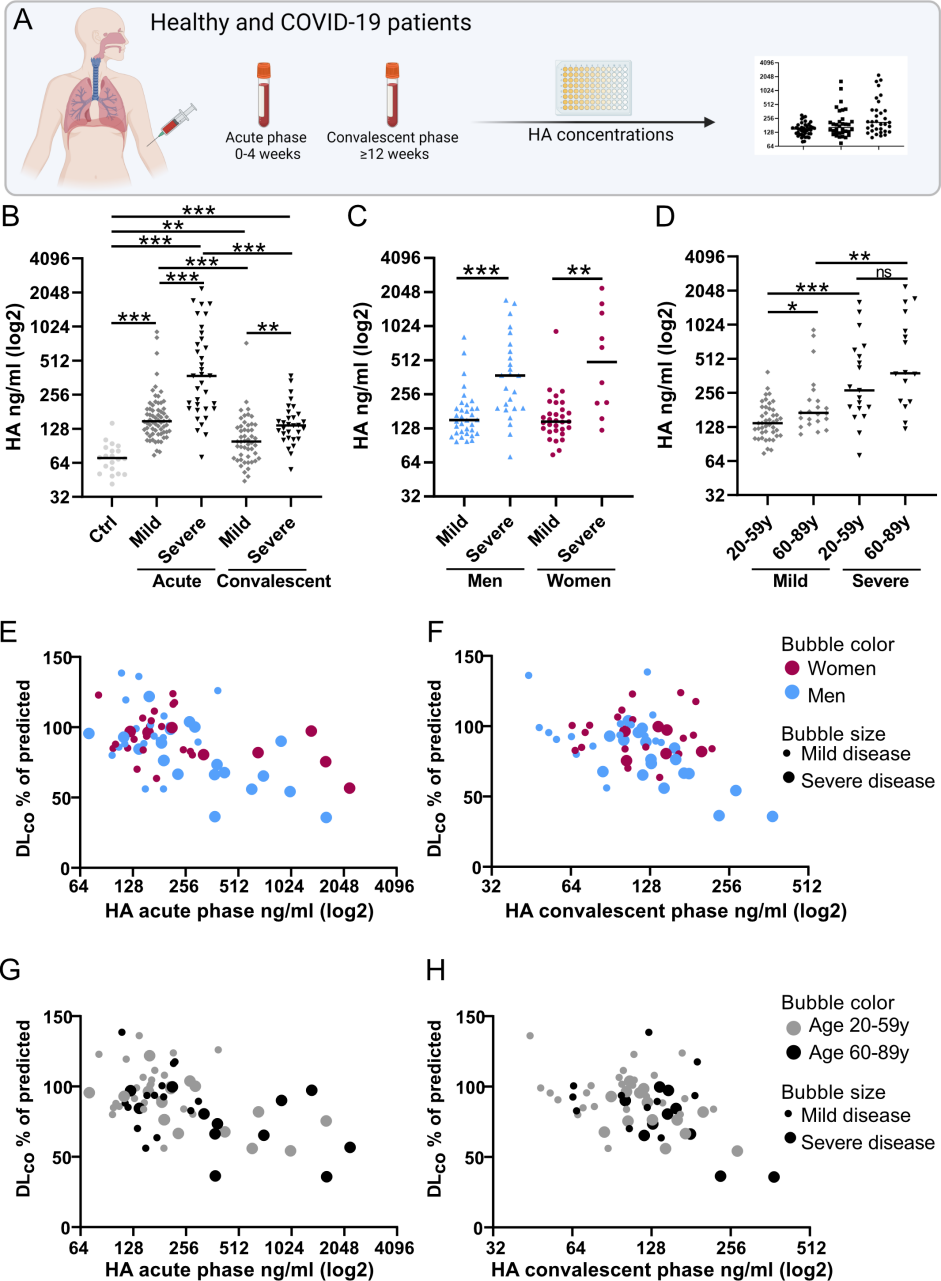
High concentrations of hyaluronan in plasma are associated with COVID-19 severity and long-term lung impairment. (**A**) Schematic overview of the collection of patient samples for HA analysis. (**B**) HA concentrations in plasma from patients with severe and mild COVID-19 compared to controls (Ctrl). Samples were taken during the acute phase (0–4 weeks from disease onset) and again during the convalescent phase of the disease (≥12 weeks). HA concentrations were determined by ELISA. (**C and D**) HA concentrations in plasma during the acute phase grouped based on severity and (C) sex or (D) age. Each dot represents one patient, and the line represents the median. Severity was based on the WHO Clinical Progression Scale with patients requiring high-flow nasal oxygen treatment and/or admission to the intensive care unit during the acute phase of illness classified as “severe,” corresponding to WHO-CPS 6–9, and all other patients as “mild,” WHO-CPS 1–5. Statistical significance was calculated using the Kruskal-Wallis test followed by Dunn’s *post hoc* test (*P < 0.05, **P < 0.01, and ***P < 0.001). (**D and E**) The percentage of predicted values of diffusion capacity (DL_CO_) related to HA plasma concentrations in (E) the acute phase or (F) the convalescent phase divided by sex and disease severity. (**G and H**) The percentage of predicted values of DL_CO_ related to HA plasma concentrations in (E) the acute phase or (F) the convalescent phase divided by age and disease severity. Multiple linear regression analysis including independent variables: Sex, chronic lung disease, cardiovascular disease, hypertension, diabetes, smoking (current or previous), obesity (BMI ≥ 30), severity of COVID-19 (mild or severe), and age (20–59 and 60–89 years) showed a significant negative correlation between HA levels in both the convalescent phase (β = −9.35, *P* = 0.033) and acute phase (β = −6.0, *P* = 0.047) and DL_CO_%pred.

The concentration of HA in plasma samples was determined using enzyme-linked immunosorbent assay (ELISA) in order to assess the association between HA and disease severity. A general increase in HA concentrations was observed in samples taken during the acute phase of the disease in both patients with mild and severe COVID-19, as compared to healthy controls (Fig. 3B). The HA concentrations were even further increased in patients with severe COVID-19. Interestingly, although HA concentrations declined in the convalescent phase (mild *P* ˂ 0.0001, severe *P* ˂ 0.0001), they remained elevated compared to healthy controls, especially in patients with severe disease. A sex comparison of HA plasma concentrations in the acute phase showed a similar increase in HA in severe COVID-19 in women and men (Fig. 3C). However, the HA levels remained higher among women compared to men (*P* = 0.043, data not shown) in the convalescent phase of mild COVID-19. Analyses assessing the impact of age showed an increase in HA concentrations in severe compared to mild COVID-19 in both older (60–89 years) and younger (18–59 years) patients, with a general trend toward higher HA concentrations in the older age group (mild disease: *P* = 0.01, severe disease: *P* = 0.17) (Fig. 3D). As we have seen that HA accumulated in the lungs during COVID-19, and systemic levels of HA remain high even after 12 weeks, we next wanted to investigate if systemic HA was associated with actual lung function. HA levels of acutely infected patients were associated with the percentage of predicted diffusion capacity (DL_CO_%pred) 3–6 months after infection (Fig. 3E through H). Lung function measurements were available in 70 out of 103 patients. Multiple linear regression models were constructed, including sex, chronic lung disease, cardiovascular disease, hypertension, diabetes, smoking (current or previous), obesity (BMI ≥ 30), severity of COVID-19 (mild or severe), and age (20–59 and 60–89 years) as independent variables to adjust for these common confounders. Our results showed a negative correlation between HA levels in both the convalescent phase (β = −9.35, *P* = 0.033) and acute phase (β = −6.0, *P* = 0.047) and DL_CO_%pred (Fig. 3E through H; Tables S1 and S2).

In summary, plasma concentrations of HA were substantially increased in COVID-19 patients and correlated to disease severity and reduced diffusion capacity. We next performed infection experiments in a human 3D-lung model to further study the potential mechanisms behind the increase of HA in COVID-19.

### SARS-CoV-2 infection causes an inflammatory response, which is counteracted by corticosteroids in a human 3D-lung model

**Fig 4:**
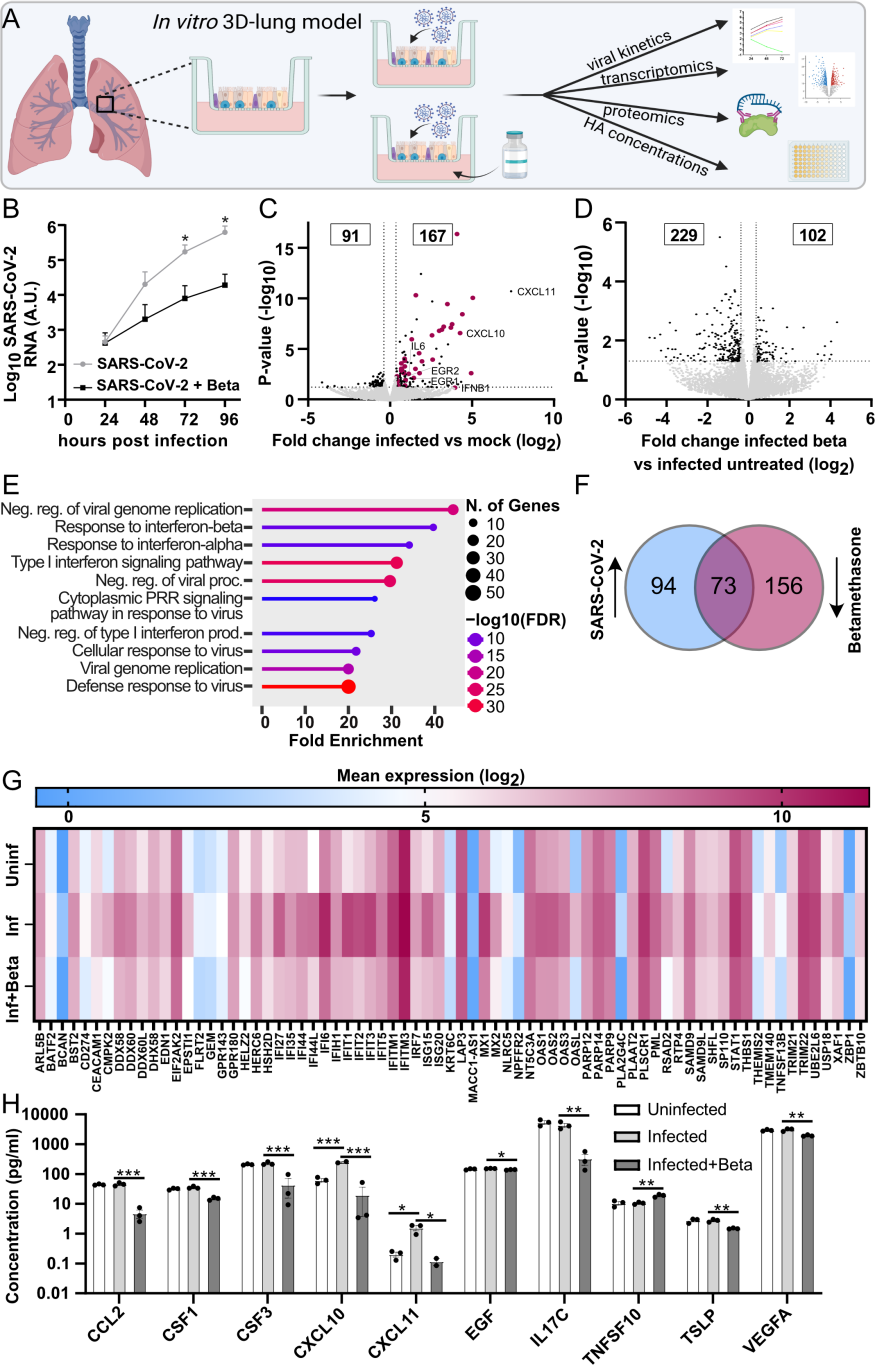
Inflammatory response and effect of corticosteroid treatment in a SARS-CoV-2-infected 3D-lung model. (**A**) Schematic overview of the primary 3D-lung model that was infected with SARS-CoV-2 in the presence or absence of corticosteroids to investigate the effect on inflammation and hyaluronan metabolism. (**B**) The lung cultures based on differentiated primary human bronchial epithelial cells at an air-liquid interface were treated with/without betamethasone (Beta) in the basal media and infected with SARS-CoV-2 (multiplicity of infection = 0.5, *n* = 3). The accumulated viral release from the apical side of the cultures was quantified by qPCR at indicated time points. Statistical significance was calculated by unpaired *t*-test (*P < 0.05). (**C and D**) Volcano plot showing differentially expressed genes between (C) infected vs uninfected (mock) lung cultures and (D) infected lung cultures with betamethasone vs without betamethasone treatment (*n* = 3). The statistical *P*-value (-log_10_) is plotted against the fold change in gene expression (log_2_). Dotted lines highlight the significance cut off corresponding to a fold change of 1.3 and *P*-value = 0.05. Genes involved in defense response to virus (GO:0051607) are highlighted in panel C. (**E**) Gene ontology enrichment of biological processes among the 167 genes significantly upregulated by SARS-CoV-2 infection. (**F**) Venn diagram showing the overlap between the 167 genes significantly upregulated upon SARS-CoV-2 infection compared to 229 genes downregulated by betamethasone treatment. (**G**) Heatmap displaying the mean expression (log_2_) of the overlapping genes upregulated by SARS-CoV-2 infection and downregulated upon betamethasone treatment in uninfected, infected, and infected + betamethasone-treated lung cultures. (**H**) Cytokine levels in basolateral samples from HBEC ALI cultures collected at 96 h post-infection analyzed by proximity extension assay (*n* = 3). Mean values and SEM are shown; statistical significance was calculated using a one-way ANOVA followed by Tukey’s *post hoc* test (*P < 0.05, **P < 0.01, and ***P < 0.001).

We used a lung model that closely resembles the human respiratory tract based on primary human bronchial epithelial cells (HBECs) isolated from human donors. This model was used to characterize the cellular pathways affected by SARS-CoV-2 infection and the effects of corticosteroid treatment with respect to inflammation and HA metabolism. The cells were differentiated at an air-liquid interface (ALI) to form a polarized epithelium containing an apical layer of fully functional secretory and ciliated cells and an underlying layer of basal cells (21). The lung cultures were treated with or without the corticosteroid betamethasone and infected with SARS-CoV-2 (Fig. 4A). The course of infection was monitored daily by the collection of apical secretions containing released progeny virus, and the viral load was quantified by qPCR. A distinct increase in viral RNA was observed over time, indicating an active viral replication in the HBEC ALI cultures (Fig. 4B). Betamethasone-treated cultures showed reduced levels of released progeny virus. The infected HBEC ALI cultures were harvested at 96 h post-infection and subjected to total RNA sequencing to identify the genes and pathways regulated by SARS-CoV-2 infection and betamethasone treatment. Statistical comparison of genes expressed in uninfected vs infected HBEC ALI cultures (Fig. 4C; Table S3) identified 167 differentially expressed genes (DEGs) that were upregulated upon SARS-CoV-2 infection. Enrichment analysis of these DEGs mainly showed an effect on antiviral mechanisms among the top 10 processes, including defense response to the virus, response to type I interferon (IFN-I), and IFN signaling (Fig. 4E; Table S4). This finding aligns well with transcriptomic analyses of lung samples from COVID-19 patients, as reported in a previous study (22), showing that our lung model can mirror key aspects of the disease process. Next, a statistical comparison of infected HBEC ALI cultures with or without betamethasone treatment was performed to evaluate the effect of betamethasone treatment. This identified 102 genes that were upregulated, and 229 genes that were downregulated in the betamethasone-treated cultures (Fig. 4D; Table S3). Interestingly, 73 of the 167 genes upregulated by infection (Fig. 4C) were downregulated by betamethasone treatment (Fig. 4D), as shown by the overlapping genes in a comparison of DEGs from the two groups (Fig. 4F). A heatmap displaying the mean expression values of the individual overlapping genes showed a clear upregulation of these genes by SARS-CoV-2 infection, which was counteracted by betamethasone treatment (Fig. 4G). Several of the affected genes were involved in inflammatory responses, correlating well with the anti-inflammatory activity of betamethasone. We also collected basolateral samples from the HBEC ALI cultures at 96 h post-infection and analyzed these with a targeted cytokine panel based on proximity extension assay, allowing a quantitative concentration measurement of each cytokine (Fig. 4H). Of the 10 cytokines that showed significant changes, 8 were downregulated by betamethasone treatment, but not affected by the infection. CXCL10 and CXCL11, two chemokines important for the recruitment of immune cells, were both upregulated by the SARS-CoV-2 infection and subsequently downregulated by steroid treatment.

### SARS-CoV-2 affects HA metabolism by upregulation of HA synthases and downregulation of hyaluronidases

**Fig 5:**
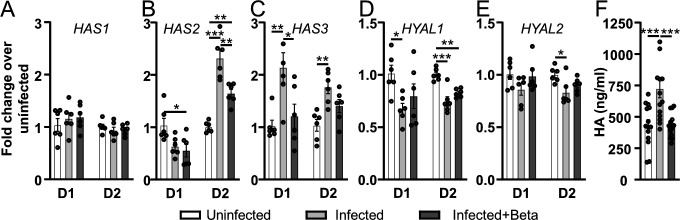
Effect of SARS-CoV-2 infection on hyaluronan synthases and hyaluronidases. The gene expression levels of HA synthases (A) *HAS1*, (**B**) *HAS2,* and (C) *HAS3* along with the hyaluronidases (D) *HYAL1* and (E) *HYAL2* were determined by qPCR 120 h post-SARS-CoV-2 infection of lung cultures from two different donors (D1 and D2, *n* = 6 per donor). (**F**) HA concentrations were determined by ELISA in apical secretions from lung cultures from the two donors 120 h post-infection (*n* = 6 per donor). Mean values and SEM are shown; statistical significance was calculated using a one-way ANOVA followed by Tukey’s *post hoc* test (*P < 0.05, **P < 0.01, and ***P < 0.001).

To further investigate the direct effect of SARS-CoV-2 on HA synthesis and degradation, expression levels of the three human hyaluronan synthases, *HAS1, HAS2,* and *HAS3,* and the two major hyaluronidases, *HYAL1* and *HYAL2*, were determined in HBEC ALI cultures from two different donors 5 days post-infection. No effect was seen on *HAS1* expression, but SARS-CoV-2 infection increased *HAS*2 in donor 2 and *HAS3* expression in both donors (Fig. 5A through C). Betamethasone treatment of infected cultures resulted in a reduction of the upregulated *HAS2* expression in donor 2 and *HAS3* in donor 1 (Fig. 5B and C). Additionally, SARS-CoV-2 infection decreased the expression of hyaluronidase *HYAL1* in both donors and *HYAL2* in donor 2 (Fig. 5D and E). The hyaluronidase genes were not significantly affected by betamethasone treatment (Fig. 5D and E). Both the upregulation of HA synthases and the downregulation of degrading hyaluronidases can induce an increase in HA concentrations. Indeed, measurements of HA concentrations in the apical secretions from HBEC ALI cultures from both donors confirmed the transcriptional changes with an increase in HA in cultures infected by SARS-CoV-2, which were kept at baseline concentrations when simultaneously treated with betamethasone (Fig. 5F).

**Fig 6:**
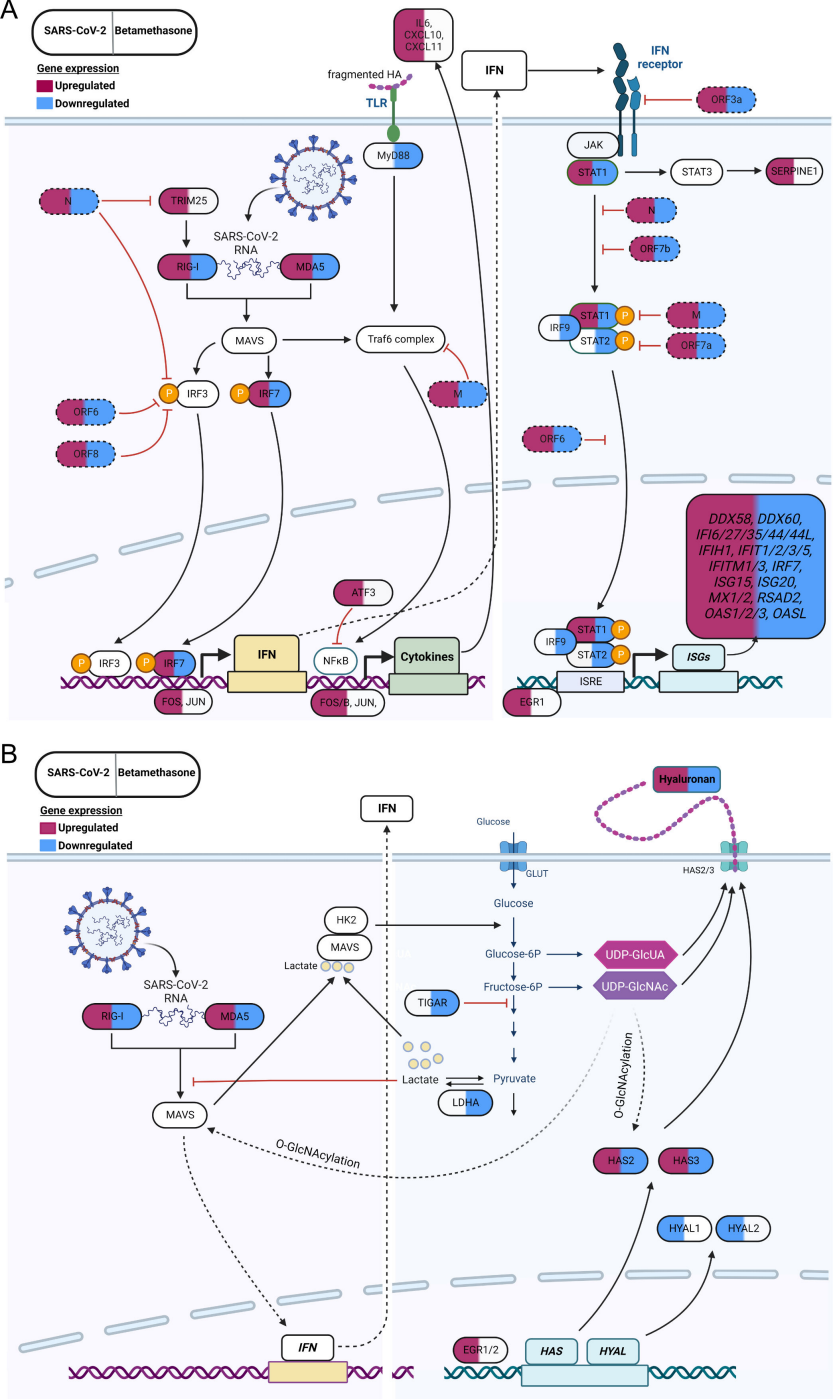
The effect of SARS-CoV-2 and betamethasone on cellular pathways regulating IFN-I responses and hyaluronan production in a human *in vitro* 3D-lung model. Schematic presentation of a cell infected with SARS-CoV-2 with or without betamethasone treatment and affected cellular pathways regulating (A) the IFN-I response and (B) HA production. The left side of the gene shows the effect on gene expression upon SARS-CoV-2 infection, and the right side shows the effect of betamethasone treatment in infected lung cultures. Purple red corresponds to an increase in gene expression, and blue indicates a decrease in gene expression. Viral genes are represented by dotted lines.

Taken together, our results identified genes involved in viral defense and inflammation affected by SARS-CoV-2 infection. Betamethasone treatment demonstrated a general counteraction against the viral transcriptional effect. SARS-CoV-2 infection and betamethasone treatment also affected genes with a potential impact on HA production and degradation. In addition to the effects on HA synthases and hyaluronidases, we observed an upregulation of transcription factors early growth response 1 and 2 (*EGR1, 2*) upon SARS-CoV-2 infection and downregulation of lactate dehydrogenase A (*LDHA*) and TP53-induced glycolysis regulatory phosphatase (*TIGAR*) after betamethasone treatment, all of which are involved in the glycolysis and therefore affect HA metabolism (Fig. 4C and D; Table S3). Based on our results, we present a model where the positive action of betamethasone in severe COVID-19 patients is a combined action of a reduced inflammatory response (Fig. 6A; Table S3) and a reduction of the pathological overproduction of HA upon SARS-CoV-2 infection (Fig. 6B; Table S3).

## DISCUSSION

We have previously shown that the lungs from deceased COVID-19 patients are filled with a clear liquid jelly consisting of HA (9), which impairs the capillary-alveolar gas exchange and leads to respiratory failure. In this study, we investigated the morphology of the lung in 3D, the presence of fragmented HA during severe COVID-19, and the correlation between systemic HA levels and diffusion capacity of the lungs after recovery. Using a human *in vitro* 3D-lung model, we also showed effects on genes involved in HA metabolism that contribute to the pathological increase, which were counteracted by corticosteroid treatment, suggesting potential new therapeutic targets.

Morphological studies of tissue are commonly done by immunohistochemistry of thin tissue slices. These have the advantage of a high resolution potentially down to the subcellular level, and many cellular markers can be used to identify different features. However, it is much more difficult to analyze the composition and morphology of lung tissue, especially the alveolar volume and surface areas. We therefore used LSFM to study the morphology of lung necropsies and biopsies in 3D. Our results showed a reduction in the number and size of the alveoli, as well as the surface area used for gas exchange at the final stage of COVID-19 compared to healthy controls. Interestingly, LSFM has been used previously in a ferret model of COVID-19, but no similar obstructions were seen in their lungs (23). With histochemistry, we showed that the alveoli were filled with HA and this HA was highly fragmented in the COVID-19 necropsies compared to healthy controls, likely contributing to the inflammatory milieu visualized by large infiltrates of neutrophils. The HA was also fragmented in active severe COVID-19 in both nasopharyngeal and endotracheal aspirates. Interestingly, hyper-induction of HA in the lungs appears, in many respects, to be a reversible process. Even though reduced diffusion capacity correlates to disease severity (18) and is related to the severity of radiologic lung involvement at admission (24), there is still restitution. This is also indicated in our data, where the recovered COVID-19 patient did not show a reduced mean alveolar surface area compared to the healthy controls. Although this provides an interesting observation, it is based on a single case and larger studies are needed to validate these results and better understand the broader implications. However, the presence of collagen coils that disrupt alveolar walls in the necropsy COVID-19 lungs suggests that the filling of HA in the alveoli precedes the development of collagen fibrosis and irreversible lung damage. This is supported by previous findings in both human inflammatory diseases and in a lung damage rat model (7, 25, 26). The role of HA size in fibrosis is not clear, but it has been demonstrated that the interaction of fragmented HA and the receptor HA-mediated motility receptor increases the infiltration of fibroblasts, leading to uncontrolled wound healing with collagen production and fibrosis (27).

We showed that despite an overall reduction in systemic HA concentrations during the convalescent phase (≥12 weeks), the plasma levels did not normalize in either mild or severe COVID-19 patients and remained significantly higher compared to the control group. Prior studies have demonstrated an elevation of HA concentration associated with disease severity in the acute phase of COVID-19 (11–14), but the long-term consequences of COVID-19 on HA levels have not previously been thoroughly investigated. The tissue half-life of HA ranges from half a day to 2–3 days, and the half-time in blood is even shorter (28). The sustained elevation of plasma HA concentrations demonstrated here, therefore, suggests an imbalance in the production and degradation of HA that remains for at least 12 weeks after disease onset, even after mild disease. The correlation of high plasma levels of HA in both acute and convalescent phases with reduced diffusion capacity also implies that plasma HA may be used as a predictive biomarker for future lung function impairment.

The molecular mechanisms behind the observed increase in HA synthesis upon SARS-CoV-2 infection remain to be clarified. Based on the results of this study, we here propose a model in which SARS-CoV-2 infection causes transcriptional changes of genes involved in HA metabolism, which are partially counteracted by corticosteroid treatment. In our human *in vitro* 3D-lung model, SARS-CoV-2 infection caused an upregulation of the transcription factors *EGR1* and *EGR2*, and we hypothesize that this in turn may activate the expression of HA synthases *HAS2* and *HAS3*. At the same time, SARS-CoV-2 infection decreased the degrading hyaluronidases. We observed donor variance in the expression of both hyaluronan synthases and hyaluronidases upon SARS-CoV-2 infection, likely due to genetic differences and individual immune responses. This variance highlights the complexity of HA regulation and the role of personalized factors in infection response. In addition, it is possible that other pathways can affect HA production. It was recently shown that specific RNA sequences in the SARS-CoV-2 genome can activate the expression of *HAS2* and, consequently, increase HA synthesis (29). In addition, changes to the glycolysis pathway may affect HA levels due to an increased production of HA precursor molecules (30). Others have shown that SARS-CoV-2 infection and subsequent replication induce an increased glucose metabolism in infected cells (31, 32). Corticosteroid treatment reduced the overall HA concentration in our 3D-lung model during SARS-CoV-2 infection. Interestingly, besides counteracting the SARS-CoV-2-induced effect on hyaluronan synthases, corticosteroid treatment of the infected 3D-lung model also decreased the expression of LDHA and TIGAR, which may both contribute to decreased HA production via the glycolysis pathway (33, 34). Despite the complex composition of cells normally found in the lung, our 3D-lung model lacks stromal cells and immune cells, which could also impact HA regulation and accumulation. Their combined action on HA metabolism would be an interesting subject for further studies.

Today, one of the few evidence-based treatment options for patients with severe to critical COVID-19 is corticosteroids (15). However, the timing of treatment seems to be crucial. Early treatment of COVID-19 patients with corticosteroids has been shown to have less positive effect than treating severely ill patients (15). The increased levels of HA in the blood from both mild and severe cases of COVID-19, shown by us and others (11–14), indicate a dysregulated HA metabolism in COVID-19, which was also supported by the findings in our 3D-lung model. Hyaluronan production and/or degradation, therefore, poses an attractive treatment target for severe COVID-19. Hymecromone, an FDA-approved drug for the treatment of biliary spasms, has a more direct action against HA synthesis (35) and has been safely shown to specifically reduce HA levels in healthy participants (36). Such a high-precision treatment could potentially avoid some of the negative effects of corticosteroids and enable treatment at an earlier stage to prevent disease progression. A recent clinical trial with hymecromone showed efficient inhibition of COVID-19 progression and warrants further investigations (37). It is also noteworthy that elevated HA levels are associated with disease severity in newer SARS-CoV-2 variants (14), and even non-SARS-CoV-2 lung infections (14), suggesting that treatments targeting HA production could be of relevance for multiple types of lung infections.

In summary, our results showed destructed lung morphology with markedly decreased gas exchange surface in COVID-19, fragmented inflammatory HA in both nasopharyngeal aspirate and endotracheal aspirate, and sustained increased levels of HA in peripheral blood in COVID-19 patients. The increased HA expression could be explained by the mechanisms identified in our *in vitro* lung model including an imbalance in HA production and degradation induced by SARS-CoV-2 infection. We also show that elevated systemic HA levels both in acute COVID-19 and convalescence phase negatively correlate with diffusion capacity in the lungs during convalescence. Further studies are needed to evaluate systemic HA as a biomarker for long-term lung function impairment. We show that HA is an important factor in COVID-19 pathogenesis, and studies on targeting HA metabolism as an alternative or complementary treatment to corticosteroids to reduce the acute and long-term health consequences of COVID-19 are warranted.

## MATERIALS AND METHODS

### Tissue pre-processing for optical 3D-imaging

Lung biopsies from four healthy controls, three deceased COVID-19 patients, and one patient who recovered from COVID-19 were washed in PBS, fixed in formalin overnight, dehydrated successively into methanol (MeOH) (25%, 50%, 75%, and 100%, 15 min each step) and stored at −20°C until use. Prior to clearing, the lung samples were treated in bleaching solution (H_2_O_2_:dimethyl sulfoxide:MeOH, 3:1:2) overnight at room temperature (RT). The bleaching solution was refreshed, and the samples were incubated for an additional 6 h at RT. Following bleaching, the samples were rehydrated into PBS (25%, 50%, 75%, and 100%, 15 min each step) and mounted in 1.5% low-melting point agarose (Lonza SeaPlaque Agarose, Lonza, USA). After solidifying overnight, the agarose pieces were cut into cuboids and dehydrated into MeOH (25%, 50%, 75%, and 100%, 15 min each step). The samples were subsequently washed in MeOH for 2 × 24 h on rotation in order to remove any residual water. Following MeOH washes, the samples were optically cleared in BABB (benzyl alcohol, benzyl benzoate, 1:2) changing the solution every 12 h until the biopsies were completely cleared.

### Light sheet fluorescence microscopy and 3D image processing

High-resolution 3D images of the lungs were acquired using an UltraMicroscope II (Miltenyi Biotec, Germany) fitted with a 1× Olympus objective (Olympus PLAPO 2XC) and a lens-corrected dipping cap MVPLAPO 2× DC DBE objective attached to an Olympus MVX10 zoom body with a 3,000 step chromatic correction motor. The lung regions of interest were captured at a magnification of 1.6× with a scan depth of 1,000 µm, a dynamic focus range across the specimen capturing 10 images per section, and a step-size of 5 µm yielding a voxel size of 1.89 × 1.89 × 5 µm. The samples were scanned for autofluorescence with the filters Ex 470/40, Em: 525/50 using an exposure time of 300 ms. Optical sections were saved in *ome.tif format native to the ImspectorPro software (version 7.0.124.9 LaVision Biotex GmbH, Germany). The *ome.tif files were converted into 3D projection *ims files using the Imaris file converter (version 9.9.1, Bitplane, UK)

3D volumes were generated using the built-in surfacing method of Imaris (version 9.8.0, Bitplane, UK). To quantify the “empty space volume,” roughly translating to the alveolar volume, the threshold was set to only include hypointense space using absolute intensity for surfacing, with a surface grain size set to 0.8 µm. Furthermore, an exclusion filter removing objects consisting of less than 10 voxels was applied to remove general noise. The anatomy surface was generated and set to include all signals above the hypointense signal with a surface grain size of 3.78 µm and a 10-voxel exclusion filter. Surfaces generated from hypointense regions outside the tissue volume were manually excluded when possible. Volumes and area of the segmented surfaces were extracted from Imaris as Excel (Microsoft, Office 365, version 2301) *.XML file format for quantification. Statistical analysis was done using GraphPad Prism (LCC, version 9.5.0).

### Staining of lung biopsies

#### Staining for hyaluronan with hyaluronan binding probe

After deparaffinization and rehydration, the sections were incubated with a solution of 3% H_2_O_2_ in methanol for 5 min, washed once in distilled water, once in PBS, and incubated with bovine serum albumin (10 mg/mL) for 30 min to block nonspecific binding sites. All slides were then washed in PBS. Control slides were preincubated with *Streptomyces* hyaluronidase 50 units/mL (Sigma), a selective carbohydrate-digesting enzyme, for 4 h at 37°C. This enzyme specifically degrades HA and therefore serves as a control, showing the specificity of the method. The slides were next washed twice in PBS and incubated with a biotin-marked HA binding protein (1:40 dilution) at 4°C overnight. After being washed 2 × 10 min in PBS and incubated with Vectastain-Elite Avidin-Biotin complex reagent (Vector Laboratories) for 40 min, the sections were washed 3 × 10 min in PBS and incubated for 5 min in a solution of 3,3′-diaminobenzidine (Vector Laboratories). Following a wash in tap water for 5 min, the sections were counterstained with Mayers hematoxylin, washed again in tap water, and finally dehydrated and coverslipped.

#### Masson trichrome staining

Sections were placed in xylen for deparaffinization before rehydration in ethanol. The sections were flushed in tap water before fixation at RT in Bouin’s solution overnight. The fixative was removed from the sections with tap water before staining the cell nuclei with Weigert’s hematoxylin for 5 min. The sections were flushed for 5 min with tap water for the removal of excess dye, followed by a rinse in distilled water. Biebrich Scarlet-Acid Fuchsin stained the cytoplasm and collagen of the lung tissue for 5 min, and the sections were then washed with distilled water. Collagen was decolorized with phosphomolybdic-phosphotungstic acid solution from Trichome stain (Masson) kit (Sigma-Aldrich) for 5 min. Then, the collagen was stained for 5 min with aniline blue, followed by 2 min in 1% acetic acid. The sections were flushed in tap water and then dehydrated in two rinses in 70% ethanol, 2 × 5 min 96% ethanol, and 2 × 5 min 99.5% ethanol. Finally, the cuts were set in the xylene, 2 × 8 min, before the cover glass (Knittel Gläser) was mounted with DPX (Sigma-Aldrich).

#### Scanning of slides

Slides were scanned with PANNORAMIC 250 Flash III (3DHISTECH) using the software Pannoramic Scanner Software version 3.03 (3DHISTECH). The scanned slides were evaluated in SlideViewer version 2.5 (3DHISTECH).

### Isolation and fragmentation of HA from lung biopsies and aspirates

#### HA isolation

Cellular secrets were dried in a Savant SpeedVac DNA 110 vacuum concentrator (Thermo Fisher Scientific, MA, USA). Proteins and nucleic acids were digested with proteinase K (Sigma-Aldrich, St. Louis, MO, USA) and benzonase nuclease (Sigma-Aldrich) on two consecutive days. At the end of each digestion, chloroform was added to each sample, and the extracted aqueous phase was solvent exchanged to 0.1 M NaCl using Amicon Ultra 3K concentration units (Millipore, Billerica, MA, USA) followed by overnight precipitation in 99% ethanol. Sulfated glycosaminoglycans and remaining non-HA contaminants were removed with anion-exchange mini spin columns (Thermo Fisher Scientific, MA, USA), based on NaCl binding. Finally, to remove salt, the sample was solvent exchanged to 20 mM ammonium acetate (pH 8.0) in Amicon Ultra 3K concentration units.

#### HA molecular mass analysis

HA mass analysis was undertaken using a gas-phase electrophoretic mobility molecular analysis (TSI Corp., MN, USA). The molecule diameter analyzed in the GEMMA was converted to molecular mass by analyzing HA standards ranging from 30 to 2,500 kDa (Hyalose, OK, USA). The area under the curve corresponds to the number of molecules in GEMMA. The counts were normalized to the amount of input material, measured in milliliters for NPA and ETA and in milligrams of dry weight for lung biopsies. In Fig. 2D, the average counts from the five patients in each group were calculated and used for representation.

### Study design and study population

Data and clinical samples were obtained from the CoVUm study, a prospective, multicenter observational study of COVID-19 including patients from Umeå and Örebro, and coordinated from Umeå University, Sweden (www.clinicaltrials.gov identifier NCT 04368013). The study protocol and cohort have been described in detail earlier (38). Non-hospitalized patients aged ≥15 years and hospitalized patients aged ≥18 years with a positive PCR test for SARS-CoV-2 were enrolled in the study. Written informed consent was obtained from all participants or their next of kin before the first sampling time point. At data export on 4 May 2022, a total of 543 participants, enrolled between 27 April 2020 and 28 May 2021, had been registered in the CoVUm database. Seven of the participants were excluded since they did not fulfill the inclusion criteria (false-positive PCR tests for SARS-CoV-2). Out of the remaining 536 participants, all patients classified as severely ill during the acute phase of the disease, and with available blood samples at the time of analysis, were selected for this study. In addition, 66 patients with mild COVID-19 were selected from the remaining study cohort. Participants were classified as “severe” if they required high-flow nasal oxygen treatment and/or were admitted to the ICU during the acute phase of illness, corresponding to WHO Clinical Progression Scale 6–10 (20). All other participants were classified as “mild,” corresponding to the WHO-CPS 1–5. Blood samples were obtained at the acute phase (0–4 weeks after onset) and convalescent phase (≥12 weeks). For the study outline, see Fig. 2A. A healthy control group, consisting of plasma samples collected from anonymous blood donors, was included for reference.

### Data collection and clinical samples

All clinical metadata including age, sex, Charlson Comorbidity Index (39), tobacco use, medication at enrollment, body mass index, type of respiratory support, and medical treatment were collected and managed using the REDCap electronic data capture tools hosted at Umeå University (40, 41). Clinical chemistry data of conventional inflammatory markers were extracted retrospectively from the patients’ electronic medical records. The highest and lowest values of each biomarker from each individual, during the first 180 days after symptom onset, were extracted for further analysis. Plasma samples used for HA analysis were collected at each time point in 6 mL EDTA tubes (BD Diagnostics).

### Measurement of hyaluronan concentration in plasma samples

Plasma hyaluronan concentrations were measured with a competitive HA-binding protein-based ELISA-like concentration measurement kit (K-1200; Echelon Biosciences Inc., Salt Lake City, UT, USA), according to the manufacturer’s instructions. Samples were run in duplicate, and a coefficient of variation < 10% was considered acceptable. Absorbance was measured on a ThermoMultiskan Ascent (Thermo Fisher Scientific, MA, USA) and plotted by polynomial regression against the concentration of the standard curve.

### Lung function tests

Lung function tests were conducted 3–6 months after study enrollment for non-hospitalized patients or discharge from the hospital for patients hospitalized during the acute phase of illness, as previously described (18). Reference values for DL_CO_ were calculated using The Global Lung Function Initiative Network guidelines (42).

### Assessment of hyaluronan metabolic pathways upon SARS-CoV-2 infection in an *in vitro* 3D-lung model

#### Generation of the human primary 3D-lung model

Primary human bronchial epithelial cells were isolated from proximal airway tissue obtained with informed consent from two patients, who underwent thoracic surgery at the University Hospital, Umeå, Sweden. HBECs were grown and differentiated at an air-liquid interface forming an *in vitro* 3D-lung model as previously described (21). In short, HBECs were grown and differentiated on 6.5 mm semipermeable transwell inserts (0.4 µm Pore Polyester Membrane Insert, Corning), and after 2 weeks at ALI, the cultures reached full differentiation, which was assessed using light microscopy focusing on epithelial morphology, presence of ciliated cells, and mucus production along with immunofluorescence staining for ciliated cells (acetylated-tubulin, T6793, Sigma) and goblet cells (muc5AC, Ab-1 [45M1], #MS-145-P, ThermoFisher).

#### Betamethasone treatment

Fully differentiated HBEC-ALI cultures were either mock treated or treated with betamethasone (Alfasigma) 20 h prior to infection by the addition of 700 µL fresh basal media containing 0.3 µM betamethasone. Betamethasone was selected to align with the corticosteroid treatment administered to the patients in this study. For the study outline, see Fig. 4A. Shortly before infection, the basal media were exchanged once more, and betamethasone was replenished in the treated wells. Betamethasone was replenished every 24 h post-infection.

#### SARS-CoV-2 infection

The clinical isolate SARS-CoV-2/01/human/2020/SWE (GenBank accession no. MT093571.1) was kindly provided by the Public Health Agency of Sweden. Vero E6 cells were cultured in Dulbecco’s modified Eagle’s medium (DMEM; Sigma) supplemented with 5% FBS (HyClone), 100 U/mL penicillin, and 100 µg/mL streptomycin (PeSt; HyClone) at 37°C in 5% CO_2_. Propagation of the virus was done once in Vero E6 cells for 72 h, and titration was done by plaque assay. The apical side of the HBEC ALI cultures was rinsed three times with warm PBS shortly before infection. 1.5 × 10^4^ plaque-forming units of SARS-CoV-2 was added to the apical compartment in a total volume of 100 µL infection medium (DMEM/PeSt), corresponding to an approximate multiplicity of infection of 0.05. The HBEC ALI cultures were incubated at 37°C and 5% CO_2_ for 2.5 h before the inoculum was removed, and the cultures were washed with PBS to remove the residual medium.

#### Sample collection

Accumulated progeny virus and secretions were collected from the apical side of the HBEC ALI cultures every 24 h by the addition of 100 µL warm PBS to the apical chamber followed by a 1-h incubation at 37°C and 5% CO_2_. The collected samples were stored at –80°C until RNA extraction. The progression of the infection was monitored for 4 days (96 h post-infection).

#### Virus quantification by qPCR

Viral RNA secreted from HBEC ALI cultures was extracted from 50 µL of the apical samples using the QIAmp Viral RNA kit (Qiagen) following the manufacturer’s instructions, and cDNA was synthesized from 10 µL of eluted RNA. RT-qPCR for SARS-CoV-2 RNA was performed in duplicates on a StepOnePlus Real-Time PCR System (Applied Biosystems) using the qPCRBIO Probe Mix Hi-ROX (PCR biosystems) and primers (forward: GTCATGTGTGGCGGTTCACT, reverse: CAACACTATTAGCATAAGCAGTTGT) and probe (CAGGTGGAACCTCATCAGGAGATGC) specific for viral RdRp.

### Transcriptomics total RNA sequencing

At 96 h post-infection, the mock-treated (*n* = 3), infected (*n* = 3), and infected + betamethasone-treated (*n* = 3) HBEC ALI cultures were washed three times on both sides with PBS. The HBEC ALI cultures were then lysed, and RNA extraction was done using the NucleoSpin RNA II kit (Macherey-Nagel) following the manufacturer’s instructions. RNA-seq libraries were prepared using the Smart-seq2 method and sequenced on an Illumina NextSeq 500 (75PE, v2.5 High Output kit). STAR 2.7.1a was used to align the reads against a reference genome consisting of GRCh38 and Sars_cov_2.ASM985889v3. A gene expression table was produced using featureCounts (43). Differential expression analysis was performed using DESeq2 (44). Differentially expressed genes were defined by a fold change > 1.3 and *P* value < 0.05. Gene ontology enrichment analysis for biological processes was done with the 167 DEGs upregulated by SARS-CoV-2 infection using Shiny GO (V 0.77, http://bioinformatics.sdstate.edu/go) (45) applying standard settings with no background uploaded and FDR set to 0.001. Pathways were selected by FDR and sorted by fold enrichment, and the 10 top are displayed in Fig. 4E.

### Cytokine quantification

At 96 h post-infection, samples were collected from the basal chambers of each of the HBEC ALI cultures. The samples were inactivated with Triton X-100 at a final concentration of 1% followed by incubation at room temperature for 3 h. The levels of 45 cytokines were quantified by Proximity Extension Assay (Olink Target 48 Cytokine panel at Affinity Proteomics Uppsala, SciLifeLab Sweden), which gives absolute (pg/mL) and relative (normalized protein expression) concentration measurements of 45 pre-selected cytokines.

### Quantification of HA synthases and hyaluronidases by qPCR

Total RNA from cells was extracted using the Nucleo-Spin RNA II kit (Macherey-Nagel). One thousand nanograms of RNA was used as an input for cDNA synthesis using High-capacity cDNA Reverse Transcription Kit (Thermo Fisher). Cellular HA synthases (*HAS1, 2,* and *3*) and hyaluronidases (*HYAL1* and *2*) were quantified using qPCRBIO SyGreen mix Hi-ROX (PCR Biosystems) and QuantiTect primer assay (Qiagen, *HAS1*;QT02588509, *HAS2*;QT00027510, *HAS3*;QT00014903, *HYAL1*;QT01673413, and *HYAL2*;QT00013363) with actin as a housekeeping gene (QT01680476) and run on a StepOnePlus Real-Time PCR System (Applied Biosystems).

### Measurement of HA concentration in apical secretions from HBEC ALI cultures

Apical secretions from HBEC ALI cultures were collected at 120 h post-infection, and HA concentrations were measured with a Hyaluronic Acid AlphaScreen Assay (K-5800; Echelon Biosciences Inc., Salt Lake City, UT, USA), according to the manufacturer’s instructions. AlphaScreen beads from the Histidine (Nickel Chelate) Detection Kit (PerkinElmer, MA, USA) were used. Chemiluminescent emission was measured on a SpectraMax i3x (Molecular Devices, CA, USA) and plotted by polynomial regression against the concentration of the standard curve.

### Statistical analysis

Statistical analysis was performed with Graphpad Prism 9 and Jamovi version 2.2.5 (The jamovi project [2021]) and jamovi version 1.6) (retrieved from https://www.jamovi.org). Descriptive variables of the patient cohort were analyzed by Mann-Whitney *U*-test (continuous variables) and Fisher’s exact test or chi-square test (dichotomous variables). Missing data were handled by complete case analysis. No correction for multiple testing was performed. Multiple linear regression was used to explore the association between DL_CO_ and HA levels in plasma during the acute illness and the convalescent phase. Independent variables were sex, chronic lung disease, cardiovascular disease, hypertension, diabetes, smoking (current or previous), obesity (BMI ≥ 30), severity of COVID-19 (mild or severe), and age (20–59 and 60–89 years). Log_2_ transformation was used to address skewed data in HA.

## Data Availability

The CoVUm data cannot be made publicly available according to Swedish data protection laws and the terms of ethical approval that were stipulated by the Ethical Review Authority of Sweden. Access to data from the CoVUm database is organized according to a strict data access procedure to comply with Swedish law. For all types of access, a research proposal must be submitted to the corresponding authors for evaluation. After evaluation, data access is contingent on vetting by the Ethical Review Authority of Sweden, according to the Act (2003:460) concerning the Ethical Review of Research Involving Humans. The total RNA-Seq raw data have been deposited at ArrayExpress (accession no. E-MTAB-12368). All the R and Python code has been deposited at https://github.com/henriksson-lab/covidbeta.

## SUPPLEMENTAL MATERIAL

Supplemental material mbio.01303-24-s0001.pdf Supplemental methods, figures, tables, and movie legends.

Table S3 mbio.01303-24-s0002.xlsx Differentially expressed genes upon SARS-CoV-2 infection of a 3D lung model.

Table S4 mbio.01303-24-s0003.xlsx GO enrichment analysis for biological processes of differentially expressed genes.

Movie S1 mbio.01303-24-s0004.mp4 Light sheet fluorescent microscopy scans of lung biopsies from a healthy donor.

Movie S2 mbio.01303-24-s0005.mp4 Light sheet fluorescent microscopy scans of lung biopsies from a deceased COVID-19 patient.

Movie S3 mbio.01303-24-s0006.mp4 Light sheet fluorescent microscopy scans of lung biopsies from a recovered COVID-19 patient.

ASM does not own the copyrights to Supplemental Material that may be linked to, or accessed through, an article. The authors have granted ASM a non-exclusive, world-wide license to publish the Supplemental Material files. Please contact the corresponding author directly for reuse.
