# Supplementary material for: SARS-CoV-2 infection induces hyaluronan production in vitro and hyaluronan levels in COVID-19 patients relate to morbidity and long-term lung impairment: a prospective cohort study
Source: mBio. 2024 Sep 20;15(10):e01303-24. doi: 10.1128/mbio.01303-24 (PMC11492986; doi:10.1128/mbio.01303-24)
Supplement: Supplemental material — Supplemental methods, figures, tables, and movie legends. [file mbio.01303-24-s0001.pdf]

SUPPLEMENTARY MATERIAL

Table of content:

Supplementary Methods

Supplementary figure 1

Supplementary figure 2

Supplementary figure 3

Legend supplementary movie 1

Legend supplementary movie 2

Legend supplementary movie 3

Supplementary table 1

Supplementary table 2

SUPPLEMENTARY METHODS

Immunohistochemistry staining of neutrophils in lung biopsies

Lung sections were de-paraffinized with xylene followed by step-wise re-hydration with EtOH/PBS, permeabilized and blocked in 10% v/v Goat serum, 0.2% v/v TritonX-100, and 1% w/v bovine serum albumin in PBS for 1 h at room temperature (RT). The lung sections were immunolabelled with primary (Rabbit polyclonal anti-Neutrofil elastase (Merck 481001)) and secondary antibody (Donkey anti-rabbit Alexa Fluor^{TM}555 (ThermoFisher A-31572)) diluted in 2% v/v Goat serum and 0.5% v/v TritonX-100 in PBS for overnight at 4 °C and 1 h at RT in the dark, respectively. Confocal fluorescence microscopy was performed using a Leica SP8 Laser Scanning Confocal Microscope equipped with HC PL APO 40x/1.3 and Leica Application Suit X software (LAS X, v.3.5.5, Leica).

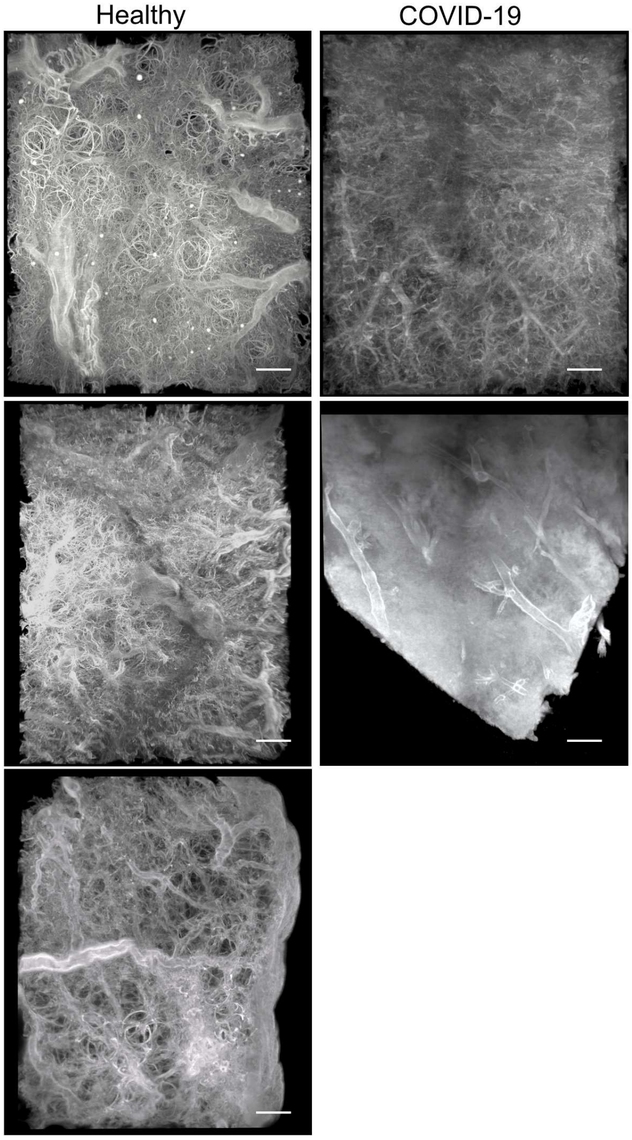

Supplementary Fig. 1. Light sheet fluorescent microscopy of biopsies from deceased COVID-19 patients and healthy donors. Maximum intensity projections of three healthy donors and two COVID-19 necropsies, scale bar is 500 µm**.**

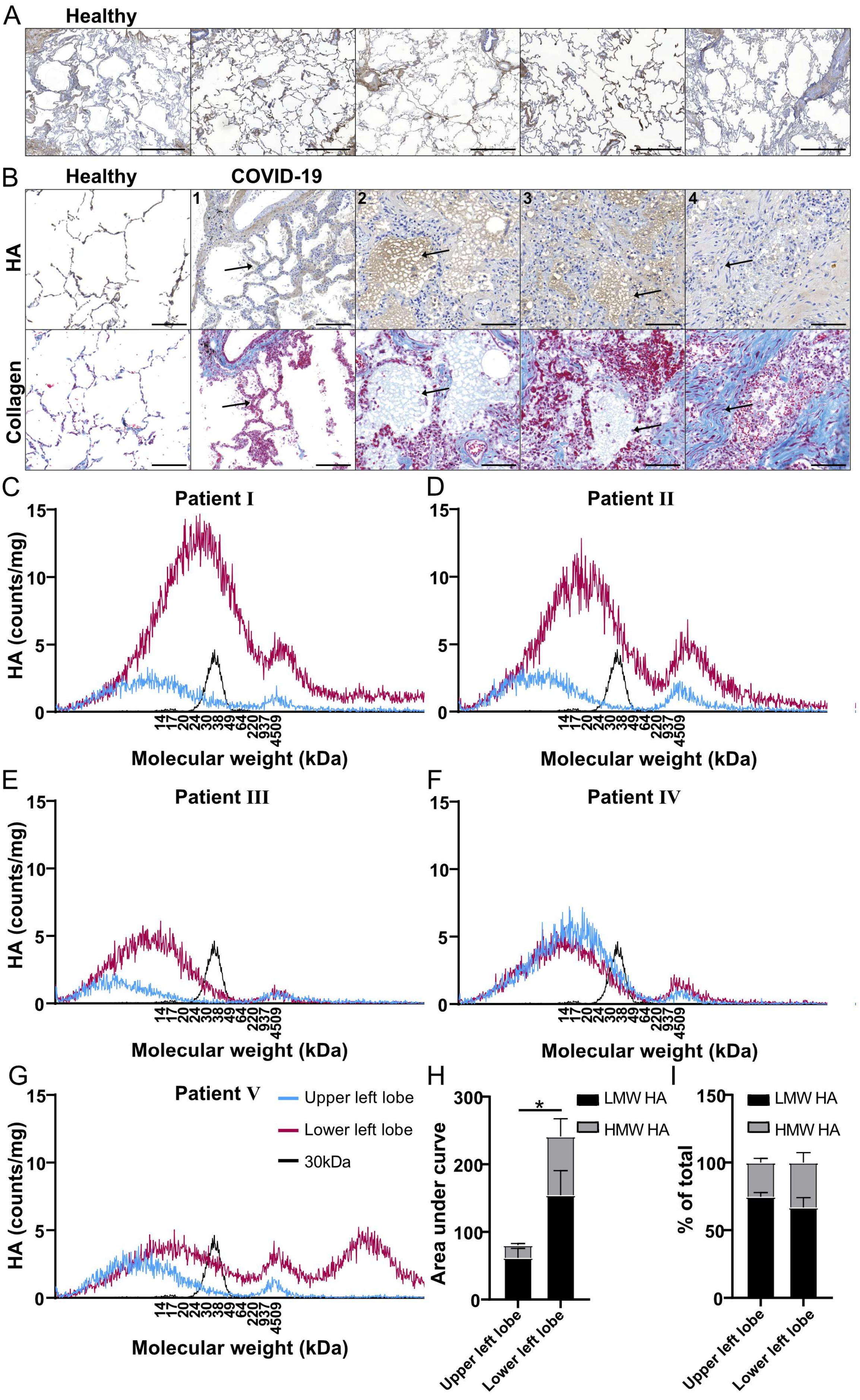

A
Healthy
B
Healthy
COVID-19
1
2
3
4
HA
Collagen
C
Patient I
D
Patient II
E
Patient III
F
Patient IV
G
Patient V
HA (counts/mg)
Molecular weight (kDa)
14
17
20
24
30
38
49
64
220
937
4509
Upper left lobe
Lower left lobe
30kDa
H
Area under curve
LMW HA
HMW HA
*
Upper left lobe
Lower left lobe
I
% of total

Supplementary Fig. 2. Hyaluronan (HA) localization and size distribution in lung biopsies. A) Lung biopsies from five healthy donors stained for HA using a HA binding protein (brown), and nuclei staining (blue). Scale bar is 1000 µm. B) Representative collagen and HA stainings in lung biopsies from healthy lung tissue and from deceased COVID-19 patients. Within the same biopsy, areas with different stages of alveolar destruction are visible, including 1) intact alveoli with enlarged walls rich in HA, 2) intact alveoli filled with HA secretions, 3) partly disrupted alveoli filled with HA secretions and 4) completely destroyed alveoli that have been replaced by collagen coils. Upper row show HA staining in brown and nuclei staining in blue, lower row show collagen staining in blue, cytoplasm in pink and nuclei in dark brown. The scale bars represent 200µm in the two left images and 100µm in the three right images. C-G) HA was isolated from the lung biopsies in Figure 2B and a size distribution analysis was performed with gas-phase electrophoretic mobility molecular analysis (GEMMA). Shown are the size distribution form the upper and lower left lobe of each patient. Low molecular weight HA with a known molecular size of 30 kDa was included as control. H) Area under curve measurements for upper and lower left lobes of patients I-V in panel C-G, as an indication of total amount of HA in each sample. Mean values and SEM are shown, statistical significance was calculated by unpaired t-test and showed a difference in amount of LMW HA, HMW HA and total amount of HA (*$p < 0.05$). I) The percentage of fragmented HA (<100kDa) in each lobe based on area under curve measurements of panel C-G. Mean values and SEM are shown, calculations with unpaired t-test did not show any significant difference.

Healthy COVID-19

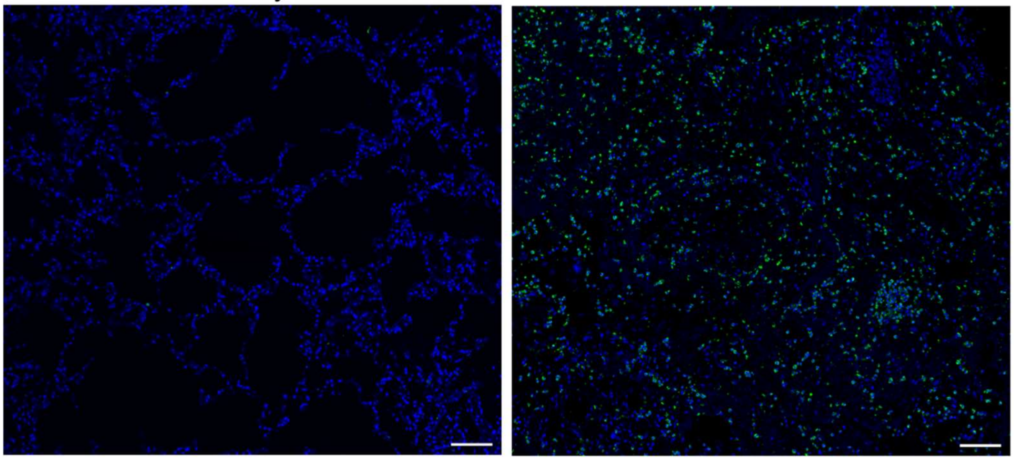

Supplementary Fig. 3. Neutrophil infiltration of COVID-19 lung necropsy. Confocal images on paraffin sections from healthy and COVID-19 lung biopsies stained with Dapi (blue) and neutrophil elastase (green). Representative pictures from imaged areas, scalebar is 100 µm.

Supplementary Movie 1. Representative movie of light sheet fluorescent microscopy scans of lung biopsies from a **healthy donor**. Initially, the anatomy (Ex 470/40, Em: 525/50) is displayed in grey as a 3D maximum intensity projection rendering, thereafter the alveolar walls are shown with a frontal optical section through the specimens, and ending with a 3D projection of iso-surfaced empty space in the lung biopsies as a proxy for alveolar volume shown in light red.

Supplementary Movie 2. Representative movie of light sheet fluorescent microscopy scans of lung biopsies from a deceased **COVID-19 patient**. Initially, the anatomy (Ex 470/40, Em: 525/50) is displayed in grey as a 3D maximum intensity projection rendering, thereafter the alveolar walls are shown with a frontal optical section through the specimens, and ending with a 3D projection of iso-surfaced empty space in the lung biopsies as a proxy for alveolar volume shown in light red.

Supplementary Movie 3. Representative movie of light sheet fluorescent microscopy scans of lung biopsies from a **recovered COVID-19 patient**. Initially, the anatomy (Ex 470/40, Em: 525/50) is displayed in grey as a 3D maximum intensity projection rendering, thereafter the alveolar walls are shown with a frontal optical section through the specimens, and ending with a 3D projection of iso-surfaced empty space in the lung biopsies as a proxy for alveolar volume shown in light red.

Supplementary Table 1. Hyaluronan in the **acute phase** as a predictor for reduced diffusion capacity.

		DL_{CO}%pred	
**Variables**	**N (%)**	***β*-coefficient (95% CI)**	**p**
**HA acute phase (log 2)**		-6.0 (-11.92 to -0.08)	**0.047**
**Sex**			
Male	41 (57.7)	ref	
Female	30 (42.3)	4.65 (-5.85 to 15.15)	0.379
**Chronic lung disease**	14 (19.7)	-3.99 (-18.3 to 10.33)	0.579
**Cardiovascular disease**	10 (14.1)	-7.46 (-23.07 to 8.15)	0.342
**Hypertension**	18 (25.3)	-7.82 (-21.54 to 5.9)	0.258
**Diabetes**	4 (5.6)	3.21 (-18.04 to 24.46)	0.763
**Smoker or previous smoker**	21 (29.6)	-2.12 (-12.98 to 8.73)	0.696
**Obesity**	24 (33.8)	-0.3 (-11.67 to 11.08)	0.959
**Severity of COVID-19**			
Mild disease	41 (57.7)	ref	
Severe disease	30 (42.3)	-7.71 (-19.86 to 4.43)	0.208
**Age groups (years)**			
20-59	44 (62)	ref	
60-89	27 (38)	-0.43 (-12.53 to 11.67)	0.944

Multiple linear regression calculated for the dependent variable DL_{CO} % of predicted (DL_{CO}%pred). Reported with β-coefficient and 95% confidence interval (CI). DL_{CO}, diffusion capacity; HA, hyaluronan; ref, reference group. Obesity defined as BMI ≥ 30. Adjusted $R^2 = 0.215$, $p = 0.008$. Significance set at $p < 0.05$.

Supplementary Table 2. Hyaluronan in the **convalescent phase** as a predictor for reduced diffusion capacity.

		**DL_{CO}%pred**	
**Variables**	**N (%)**	**β-coefficient (95% CI)**	**p**
**HA convalescent phase (log 2)**		-9.35 (-17.80 to -0.90)	**0.031**
**Sex**			
Male	41 (57.7)	ref	
Female	30 (42.3)	7.33 (-2.85 to 17.51)	0.154
**Chronic lung disease**	14 (19.7)	-4.02 (-20.42 to 12.38)	0.624
**Cardiovascular disease**	10 (14.1)	-12.07 (-28.35 to 4.21)	0.143
**Hypertension**	18 (25.3)	-6.28 (-20.45 to 7.89)	0.378
**Diabetes**	4 (5.6)	1.52 (-25.97 to 29.01)	0.912
**Smoker or previous smoker**	21 (29.6)	1.65 (-8.92 to 12.22)	0.755
**Obesity**	24 (33.8)	1.29 (-10.40 to 12.99)	0.825
**Severity of COVID-19**			
Mild disease	41 (57.7)	ref	
Severe disease	30 (42.3)	-11.77 (-22.70 to -0.833)	**0.035**
**Age groups (years)**			
20-59	44 (62)	ref	
60-89	27 (38)	1.75 (-9.82 to 13.32)	0.763

Multiple linear regression calculated for the dependent variable DL_{CO} % of predicted (DL_{CO}%pred). Reported with β-coefficient and 95% confidence interval (CI). DL_{CO}, diffusion capacity; HA, hyaluronan; ref, reference group. Obesity defined as BMI ≥ 30. Adjusted $R^2 = 0.247$, $p = 0.006$. Significance set at $p < 0.05$.
